# Genotype- and sex-dependent effects of altered *Cntnap2* expression on the function of visual cortical areas

**DOI:** 10.1186/s11689-016-9182-5

**Published:** 2017-01-19

**Authors:** Leah B. Townsend, Spencer L. Smith

**Affiliations:** 1 Neuroscience Curriculum, University of North Carolina at Chapel Hill, Chapel Hill, NC USA; 2 Department of Cell Biology and Physiology, University of North Carolina at Chapel Hill, Chapel Hill, NC USA; 3 Carolina Institute for Developmental Disabilities, University of North Carolina at Chapel Hill, Chapel Hill, NC USA; 4 Neuroscience Center, University of North Carolina at Chapel Hill, Chapel Hill, NC USA

**Keywords:** Autism, Cortical circuits, Mouse models, Higher visual areas, Sex, Intrinsic signal optical imaging

## Abstract

**Background:**

Autism spectrum disorder (ASD) is a heritable, heterogeneous neurodevelopmental disorder that is four times more likely to affect males than females. Despite this overt sex bias, it is unclear how genetic mutations associated with ASD alter cortical circuitry to produce the behavioral phenotypes by which ASD is diagnosed. Contactin-associated protein-like 2 (*CNTNAP2*) is an ASD-associated gene, and while *Cntnap2* knockout (KO) mice recapitulate many of the features of ASD, the effect on cortical circuitry is poorly understood. Moreover, although heterozygous (Het) mice are the more relevant genotype for ASD-linked *CNTNAP2* mutations in humans, to our knowledge, no effects in Het mice have been previously reported.

**Methods:**

Intrinsic signal optical imaging was used to measure functional visual responses in primary and higher visual cortical areas in male and female *Cntnap2* KO, Het, and wild-type (WT) mice. Main effect of genotype was assessed with one-way ANOVA. Visual responses were also measured in P17–18 and P30–32 KO and WT mice. Main effects of age and genotype were assessed using two-way ANOVA.

**Results:**

Visually evoked activity in dorsal stream associated higher visual areas in both KO and Het adult males was decreased relative to WT adult males. This decrease was not observed in adult females. Additionally, no significant difference was observed between WT and KO males at P17–18 with differences beginning to emerge at P30–32.

**Conclusions:**

The functional responses of cortical circuitry in male mice are more strongly affected by *Cntnap2* mutations than females, an effect present even in Hets. The observed differences in males emerge with development beginning at P30–32. These results reveal genotype- and sex-dependent effects of altered *Cntnap2* expression and can shed light on the sex-dependent incidence of ASD.

**Electronic supplementary material:**

The online version of this article (doi:10.1186/s11689-016-9182-5) contains supplementary material, which is available to authorized users.

## Background

Individuals with autism spectrum disorder (ASD) have characteristic impairments in social interaction and communication, and restricted, repetitive behaviors and interests that emerge around 2–3 years of age [1]. For reasons not fully understood, males are more likely to be affected, with the male to female incidence ratio estimated at roughly 4:1 for high-functioning individuals [2, 3]. Genome-wide association studies implicate between 400 and 1000 genes in ASD [4], making it difficult to elucidate potential shared pathological mechanisms across ASD cases. However, any molecular and cellular changes caused by these ASD-linked genetic mutations ultimately result in changes in neural circuitry [5, 6]. Thus, examining the effects of ASD-linked mutations on neuronal circuitry can inform our understanding of the pathology of ASD.

One important ASD susceptibility gene is *contactin-associated protein-like 2* (*CNTNAP2,* also known as *CASPR2*) [7, 8]. *Cntnap2* encodes a member of the neurexin superfamily that is responsible for K^+^ channel clustering in juxtaparanodes [9, 10]. Homozygous mutations in exon 22 of *CNTNAP2* result in an ASD diagnosis in 67% of cases [11], while heterozygous mutations are associated with altered brain structure and functional connectivity in otherwise neurotypical subjects [12–14]. Mouse models lacking *Cntnap2* recapitulate the hallmark features of ASD, including repetitive behaviors and impairments in social interaction and communication [15, 16]. However, the effects of this gene on the functional development of the brain remain unclear.

To examine the effects of altered *Cntnap2* expression on neural circuitry, we measured functional responses in primary (V1) and higher visual areas (HVAs) of the mouse. The cerebral cortex is critical for sensory processing and cognition, both of which are altered in ASD. In mice, cortical visual pathways diverge downstream of V1 via parallel cortico-cortical projections to multiple HVAs [17]. Analogous to the dorsal and ventral stream distinction in primates [18], mouse V1 and HVAs have distinct spatial and temporal frequency preferences [19, 20] and anatomical projections [21, 22] that support their classification into two subnetworks of cortical areas specialized for motion (dorsal stream) or form processing (ventral stream) [21, 22]. Given the impact of *Cntnap2* mutations on brain structure and functional connectivity as well as the specialization of the dorsal and ventral processing steams, we hypothesized that these subnetworks may be differentially impacted by altered levels of *Cntnap2* expression. To test this, we examined functional responses in V1 and HVAs using intrinsic signal optical imaging (ISOI) in a mouse model of *Cntnap2* mutations. Using this functional imaging technique, we have identified genotype- and sex-dependent effects of altered *Cntnap2* expression on the dorsal and ventral visual steams. These results may shed light on the phenotypic variations observed in ASD as well as the sex-dependent difference in ASD prevalence.

## Methods

### Mouse breeding

All procedures involving living animals were carried out in accordance with the guidelines and regulations of the US Department of Health and Human Services and approved by the Institutional Animal Care and Use Committee at the University of North Carolina. *Cntnap2* wild type (WT), heterozygous (Het), and homozygous (KO) mice (*n* = 7 per sex per genotype, Jackson Labs) were generated by heterozygous mating pairs with day of birth designated at P0. Mice for the P17–18 experiments (WT: males *n* = 7, KO: males = 5) were removed from the nest and immediately used for imaging experiments. Mice for the P30–32 experiments (WT: males *n* = 6, KO: males = 6) were weaned into cages of 3–5 mice and housed with all three genotypes (when possible) until used for experiments. Mice for P100 experiments were weaned into cages of 3–5 mice and housed with all three genotypes (when possible) until used for experiments between P97 and 108. Separate cohorts of adult males and females were collected. While males and females were housed separately, cages of siblings were housed beside each other to account for any environmental differences within the animal facility. Mice were raised in a temperature- and humidity-controlled room on a 12-h light/12-h dark cycle and provided ad libitum access to food and water.

### Surgical procedure

Anesthesia was induced with 5% isoflurane. This was reduced to 1.0–2.5% isoflurane for surgery and further reduced to 0.5% for imaging. After initial induction of anesthesia, 2.5 mg/kg chlorprothixene was administered (i.p.). Ophthalmic ointment (Lacri-lube, Allergan) was applied to the eyes prior to surgery and removed immediately prior to imaging. Throughout the surgery, body temperature was maintained via a heating pad. The scalp covering the right occipital cortex was resected, exposing the skull. In P17–18 *Cntnap2* WT and KO mice, the skull was then covered with physiological saline for imaging. In adult *Cntnap2* WT, Het, or KO mice, a 4-mm craniotomy was performed exposing brain and dura and then covered with physiological saline for imaging. Mice were then transferred to the intrinsic signal optical imaging (ISOI) rig. Prior to imaging, an additional dose of chlorprothixene (2.5 mg/kg, i.p.) was administered, and the mouse was maintained on 0.5% isoflurane for the duration of imaging. All procedures were performed blind to genotype.

### Imaging and visual stimuli

**Fig. 1:**
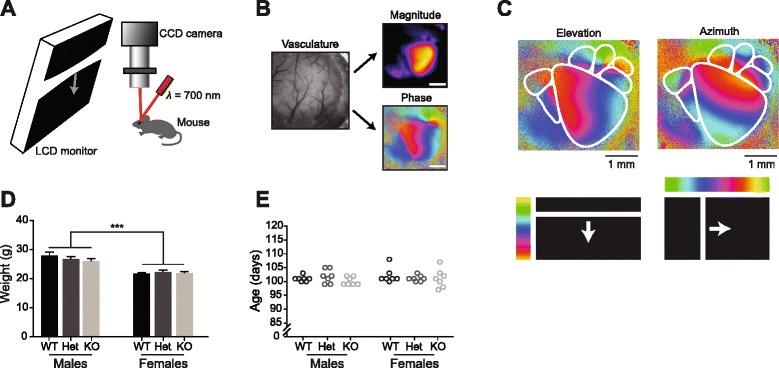
Experimental setup and baseline data. **a** Schematic of intrinsic signal optical imaging (ISOI) setup. Visual stimuli were presented to the left eye of a lightly anesthetized mouse via an LCD screen. The craniotomy over right visual cortex was illuminated with 700-nm light and changes in reflectance were captured with a CCD camera. **b** The tandem lens macroscope was focused 600 μm into the brain from the surface vasculature (*left*, shown before focusing into the brain). Magnitude and phase maps (*right*) were extracted using Fourier analysis of each pixel’s time course. *Scale bars* are 1 mm. **c** Example retinotopic maps from a male WT P101 mouse depicting six HVAs. **d** Although male mice were systematically larger, body weights were similar within sex and across genotypes (two-way ANOVA, effect of genotype *F*_(2,36)_ = 0.519, *p* = 0.6; effect of sex *F*_(1,36)_ = 54.5, *p* < 0.0001). **e** No significant differences in age were detected (two-way ANOVA, effect of genotype *F*_(2,36)_ = 0.772, *p* = 0.47; effect of sex *F*_(1,36)_ = 0.492, *p* = 0.49)

Intrinsic signal optical imaging was used to measure cortical activity [23]. The brain was illuminated with a 700-nm light and imaged with a tandem lens macroscope focused 600 μm into the brain from the vasculature (Fig. 1a). Images were acquired with a 12-bit CCD camera (Dalsa 1M30), frame grabber and custom software (David Ferster, Northwestern University with in-house modifications by Jeffrey Stirman). Images were acquired at 30 frames per second. The images with 12-bit pixel data were binned in software four times temporally and 2 × 2 spatially, resulting in images with 16-bit pixel data. From these binned images, Fourier analysis of each pixel’s time course was used to extract the magnitude and phase of signal modulation at the stimulus frequency. This in turn was used to generate magnitude maps of cortical areas modulated by the stimulus (to measure the strength of the visually evoked response) and the phase of the cortical response (to map retinotopy; Fig. 1b).

The experimenter was blind to genotype during imaging and subsequent data analysis. Visual stimuli were presented to the contralateral eye relative to the imaged hemisphere using a Dell LCD monitor (Dell U2711b, 2560 × 1440 pixels, 60 Hz) positioned 20 cm from the mouse (Fig. 1a). The monitor was tilted towards the mouse 17.5° from vertical to cover the visual field (110° by 75°) of the contralateral eye. All stimulus frames were generated and presented using MATLAB and Psychtoolbox (http://psychtoolbox.org/) [24–26] controlled by custom LabVIEW software. Retinotopy stimuli were modified to correct for the distortion caused by the flat surface of the monitor (http://labrigger.com/blog/2012/03/06/mouse-visual-stim/). Retinotopy was mapped by showing the animal a single bar drifting across the screen to identify V1 and higher visual areas (HVAs; horizontally for azimuth and vertically for elevation) (Fig. 1c). Mice were then shown a drifting grating stimulus previously shown to elicit visually evoked cortical activity modulation in V1 and HVAs [19, 20]. This drifting grating stimulus consisted of a 50° patch in the center of the visual field displaying square-wave-generated black and white bars (0.04 cycles/°) that changed in drift velocity from 0°/s (6 s) to 50°/s (2 s). This stimulus was presented for 50 8-s long cycles. On rare occasions (<5% of experiments) when a high-fidelity retinotopic map was not obtainable for a given mouse, the experiment was terminated and the data excluded from analysis.

### Data quantification and statistical analysis

Retinotopic map generation and data quantification were performed blind to genotype and sex of mice. Retinotopy maps were generated by drawing regions of interest (ROIs) offline in FIJI (Fiji is Just ImageJ; fiji.sc/Fiji)[27–29] using the magnitude and phase maps generated by the elevation and azimuth stimuli (Fig. 1c, Additional file 1: Figure S1). The borders of visual areas were identified by reversals (meridians) in the progression of retinotopy [17, 20, 23]. Once the borders of the HVAs were identified, these boundaries were used to measure the visual activity evoked in each region by the drifting grating stimulus.

To quantify the response amplitude of visually evoked activity, we analyzed the magnitude maps as follows: First, we created a duplicate copy of the magnitude map and applied a Gaussian filter (kernel size 5 × 5 pixels). This filtered map was then processed by applying a threshold to include only visually responsive cortex and converted to a mask, excluding non-responsive cortical regions. This mask was applied to the original magnitude map generated by the stimulus such that only visually responsive cortex was included in subsequent analysis. The previously defined boundaries of V1 and the HVAs were then applied to the masked magnitude map to obtain average activity measurements across each region in response to the drifting grating stimulus.

The average activation for each of these HVAs was then normalized to the average activity level of V1 for each animal. This normalization decreases the mouse-to-mouse variability in overall cortical activation and hemodynamic responses and facilitates comparison of HVA activation within individual animals.

All results are presented as mean +/− SEM. For analysis of the results from the P100 time point, a one-way ANOVA was used to assess the differences between the three genotypes (WT, Het, and KO). For analysis of the developmental data, a two-way ANOVA was used to test differences for each region between genotypes by age. Bonferroni post hoc tests were used where appropriate.

## Results

### Identification and measurement of HVA activity with ISOI

Adult mice were classified, after blind data collection and initial analysis, by genotype (WT, Het, KO) and sex. Body weights were similar within sex across genotypes (two-way ANOVA, effect of genotype *F*_(2,36)_ = 0.519, *p* = 0.60; effect of sex *F*_(1,36)_ = 54.5, *p* < 0.0001) (Fig. 1d), suggesting that loss of or reduced CNTNAP2 did not change gross physiology, consistent with prior reports [15, 16]. Adult mice were imaged between postnatal day 97 (P97) and P108 in age. No significant differences in age were present in any genotype or sex (two-way ANOVA, effect of genotype *F*_(2,36)_ = 0.772, *p* = 0.47 effect of sex *F*_(1,36)_ = 0.492, *p* = 0.49) (Fig. 1e).

In each mouse, we first used ISOI to map V1 and six HVAs: lateromedial area (LM), laterointermediate area (LI), anterolateral area (AL), rostrolateral area (RL), anteromedial area (AM), and posteromedial area (PM) using retinotopic landmarks [17, 20, 23]. All borders between cortical areas were routinely resolved, except the border between areas AM and PM (Additional file 1: Figure S1). Accordingly, areas AM and PM were combined for analysis. After mapping V1 and HVAs, we measured the magnitude of visual responses in these cortical areas.

#### Genotype and sex does not alter activity in V1 or ventral HVAs

**Fig. 2:**
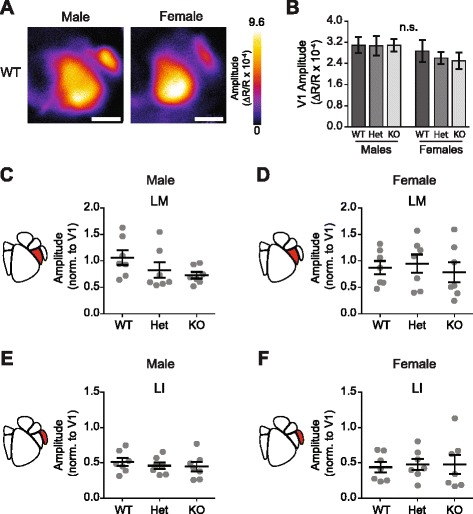
Genotype and sex does not alter activity in V1 or ventral HVAs. **a** Magnitude maps showing raw amplitude of cortical modulation in WT males and females in response to drifting grating stimulus. *Scale bar* is 1 mm. **b** Raw V1 cortical activation did not differ across sex or genotype (two-way ANOVA, effect of genotype *F*_(2,36)_ = 0.188, *p* = 0.83; effect of sex *F*_(1,36)_ = 2.73, *p* = 0.11). **c**–**f** Scatter plots of cortical modulation amplitude for ventral higher visual areas (HVA) with population mean and SEM overlaid in *black. N* = 7 mice per genotype. **c** There were no genotypic differences in cortical modulation in LM in either males (LM: one-way ANOVA *F*_(2,18)_ = 1.96, *p* = 0.17) or **d** females (LM: one-way ANOVA, *F*_(2,18)_ = 0.248, *p* = 0.78). **e** No genotypic modulation of activity was observed in LI in males (LI: one-way ANOVA *F*_(2,18)_ = 0.368, *p* = 0.7) or **f** females (LI: one-way ANOVA, *F*_(2,18)_ = 0.0510, *p* = 0.95)

In humans, heterozygous and homozygous mutations in *CNTNAP2* are associated with significant changes in structural and functional cortical connectivity [11, 12]. However, in many individuals, the effect of a heterozygous mutation does not result in an ASD diagnosis [13, 14]. To examine how differences in *Cntnap2* expression levels alter cortical area functioning, we measured the magnitude of visual responses in V1 and ventral HVAs LM and LI in adult WT, Het, and KO *Cntnap2* mice (Fig. 2). Further, given the differences in the incidence of ASD by sex, we sought to examine whether differences in *Cntnap2* expression levels have differing effects in males and females by adding a female cohort [2, 3]. Altered *Cntnap2* expression does not impact gross development or retinotopic organization given that HVAs were reliably identified in all animals, regardless of genotype or sex (Fig. 2a, Additional file 1: Figure S1). As measured with ISOI, activation of V1 did not differ by either sex or genotype (two-way ANOVA, effect of genotype *F*_(2,36)_ = 0.188, *p* = 0.83; effect of sex *F*_(1,36)_ = 2.73, *p* = 0.11; *n* = 42 animals, 7 per group) (Fig. 2b). The cortical activity of each HVA was then normalized to the average amplitude of the response in V1 (separately for each mouse) in order to account for any global variations, which can be caused by mouse-to-mouse variability or differences in anesthetic depth.

We next examined the effect of differences in *Cntnap2* expression levels on activity in LM and LI. Given the distinct visual stimulus preferences and projections of these regions, despite being grouped into the same visual pathway, we examined the effect in LM and LI separately [20–22]. We found that differences in *Cntnap2* expression levels had no effect on activity in LM in either males or females (Males LM: one-way ANOVA *F*_(2,18)_ = 1.96, *p* = 0.17; Females LM: one-way ANOVA, *F*_(2,18)_ = 0.248, *p* = 0.78; *n* = 21 measurements per HVA, 21 animals, 7 per genotype) (Fig. 2c, d). Similarly in LI, we found no effect of *Cntnap2* expression levels on visually evoked activity (Males LI: one-way ANOVA *F*_(2,18)_ = 0.368, *p* = 0.70; Female LI: one-way ANOVA, *F*_(2,18)_ = 0.051, *p* = 0.95; *n* = 21 measurements per HVA, 21 animals, 7 per genotype) (Fig. 2e, f). Taken together, this data suggests that changes in *Cntnap2* expression levels does not alter activity in primary or ventral stream-associated visual areas.

#### Genotype-dependent and sex-dependent decreases in dorsal HVAs

**Fig. 3:**
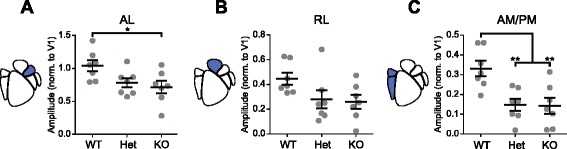
Adult males show genotype-dependent decreases in dorsal stream activity. Scatter plots of cortical modulation amplitude for dorsal HVAs in males with population mean and SEM overlaid in *black. N* = 7 mice per genotype. **a** WT mice showed higher amplitudes in AL (AL: one-way ANOVA, *F*_(2,18)_ = 4.22, *p* = 0.032). **b** WT RL trended towards increased activity (one-way ANOVA, *F*_(2,18)_ = 2.90, *p* = 0.081). **c** WT mice showed higher amplitudes in AM/PM (one-way ANOVA, *F*_(2,18)_ = 8.64, *p* = 0.0023). Results of post hoc analysis are graphically indicated where appropriate, *p < 0.05, **p < 0.01

Next, we examined the effects of changes in *Cntnap2* expression levels on dorsal steam-associated areas AL, RL, and AM/PM in both males and females. Based on previous studies identifying diverse visual stimulus preferences and projections for regions within this visual pathway, we examined the effect of *Cntnap2* expression levels on each of these regions individually [19–22]. In males, dorsal-associated area AL showed a significant difference in visually evoked activity (one-way ANOVA *F*_(2,18)_ = 4.22, *p* = 0.032; *n* = 21 animals, 7 per genotype) with KO males showing decreased activity modulation compared to WT males (*p* = 0.027) and a trend towards decreased activity modulation in Het males (*p* = 0.083) (Fig. 3a). Dorsal-associated area RL showed no difference between genotypes in visually evoked activity in response to drifting gratings (one-way ANOVA, *F*_(2,18)_ = 2.90, *p* = 0.081; *n* = 21 animals, 7 per genotype) (Fig. 3b). Dorsal-associated area AM/PM showed a significant difference in visually evoked activity modulation (one-way ANOVA, *F*_(2,18)_ = 8.64, *p* = 0.002; *n* = 21 animals, 7 per genotype) with KO and Het males showing decreased activity relative to WT controls (*p* = 0.004 and *p* = 0.005, respectively) (Fig. 3c). Taken together, this suggests that lack of *Cntnap2* expression in adult males decreases cortical activity modulation preferentially in dorsal stream HVAs relative to WT controls.

**Fig. 4:**
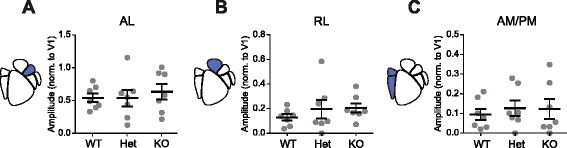
Adult females show no changes in cortical activity modulation of dorsal HVAs. Scatter plots of cortical modulation of dorsal stream HVAs in females with population mean and SEM overlaid in *black. N* = 7 mice per genotype. There were no significant differences in cortical modulation in **a** AL (one-way ANOVA, *F*_(2,18)_ = 0.262, *p* = 0.77), **b** RL (one-way ANOVA, *F*_(2,18)_ = 0.680, *p* = 0.52), or **c** AM/PM (*F*_(2,18)_ = 0.182, *p* = 0.84)

Females, however, did not show genotype-dependent decreases in dorsal stream activity modulation (Fig. 4). In contrast to males, in females, dorsal-associated areas AL, RL, and AM/PM showed no difference across genotypes in visually evoked activity in response to drifting gratings (AL: one-way ANOVA, *F*_(2,18)_ = 0.262, *p* = 0.77; RL: one-way ANOVA, *F*_(2,18)_ = 0.680, *p* = 0.52; AM/PM: one-way ANOVA, *F*_(2,18)_ = 0.182, *p* = 0.84; *n* = 21 measurements per HVA, 21 animals, 7 per genotype) (Fig. 4a–c). These results indicate that decreasing expression of *Cntnap2* in female mice does not alter visually evoked activity modulation in dorsal stream-associated higher visual areas.

### Effect of *Cntnap2* mutation in development

Given the unexpected specificity of the effects of altered *Cntnap2* expression on cortical visual circuitry, we next examined when in development the deficits observed in adult males emerge. The mammalian visual system undergoes experience-dependent maturation that relies on a sensitive or “critical” period of development for functional development [30, 31]. *Cntnap2* is expressed in mice beginning at embryonic day 14 (E14) and has many roles in cortical circuitry that change with development [10, 15, 16, 32]. As a result, it was not apparent whether the deficits observed in adult males emerge prior to the visual sensitive period (~P20–P35) as a consequence of processes that are not dependent upon experience-dependent plasticity or after this period as a result of experience-dependent refinement of this cortical circuitry [6, 15, 30, 31]. To test this, we examined visually evoked activity in HVAs in P17–18 and P30–32 WT and KO male mice in response to drifting gratings.

**Fig. 5:**
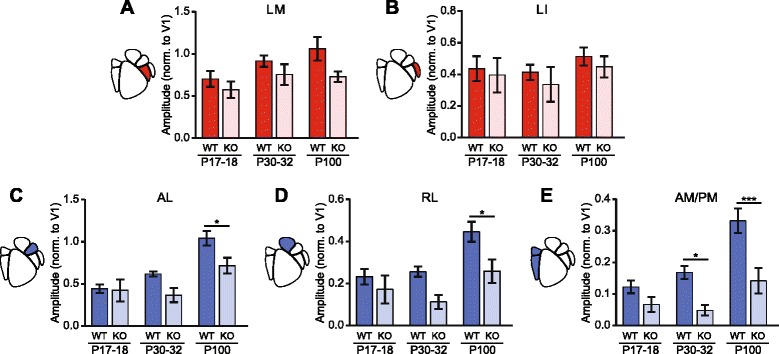
Comparison of responses of juvenile and adult males to drifting gratings. Bar plots of the developmental trajectories of cortical modulation in all observable HVAs in males. SEM overlaid in *black*. P17–18: *N* = 7 WT and 5 KO; P30–32: *N* = 6 per genotype; P100: *N* = 7 per genotype, data collected between P97 and P108. **a** There was an effect of age and genotype in LM (two-way ANOVA, age *F*_(2,32)_ = 3.40, *p* = 0.046, genotype *F*_(1,32)_ = 6.07, *p* = 0.019). **b** No differences were observed in LI. **c** AL had a main effect of age and genotype (two-way ANOVA; age: *F*_(2,32)_ = 17.2, *p* < 0.0001, genotype *F*_(1,32)_ = 17.2, *p* < 0.0001). **d** *RL* showed a main effect of age and genotype (two-way ANOVA; age: *F*_(2,32)_ = 8.05, *p* = 0.0015, genotype *F*_(1,32)_ = 12.03, *p* = 0.0015). **e** AM/PM showed both effects of age and genotype (age: *F*_(2,32)_ = 13.6, *p* < 0.0001, genotype *F*_(1,32)_ = 24.4, *p* < 0.0001). Results of post hoc analysis are graphically indicated where appropriate, *p < 0.05, ***p < 0.001

The ventral-associated HVAs, LM and LI, exhibited different patterns of functional development in response to the drifting grating stimulus. In LM, modulation of cortical activity increased with age in both WT males and KO males (two-way ANOVA, age *F*_(2,32)_ = 3.40, *p* = 0.046, genotype *F*_(1,32)_ = 6.07, *p* = 0.019) (Fig. 5a). In contrast, area LI exhibited similar cortical modulation in both genotypes at all ages examined (two-way ANOVA, age *F*_(2,32)_ = 0.912, *p* = 0.41, genotype *F*_(1,32)_ = 0.890, *p* = 0.35) (Fig 5b).

The dorsal-associated HVAs all exhibited a strong effect of development on visually evoked responses that was impaired by lack of *Cntnap2* expression. Activity modulation in area AL increased with a main effect of age (two-way ANOVA, age *F*_(2,32)_ = 17.2, *p* < 0.0001) and genotype (genotype *F*_(1,32)_ = 8.58, *p* = 0.0062) (Fig. 5c). Post hoc analysis revealed that adult WT males have significantly higher activity modulation than adult KO males in AL (*p* < 0.05) (Fig. 5c). In areas RL and AM/PM, modulation of cortical activity increased with age more in WT males than KO males (two-way ANOVAs; RL age *F*_(2,32)_ = 8.05, *p* = 0.0015, genotype *F*_(1,32)_ = 12.03, *p* = 0.0015; AM/PM age *F*_(2,32)_ = 13.6, *p* < 0.0001, genotype *F*_(1,32)_ = 24.4, *p* < 0.0001) (Fig. 5d, e). Post hoc analysis revealed that adult WT males have significantly higher activity modulation than KO males in both RL and AM/PM (*p* < 0.05 and *p* < 0.001, respectively) (Fig.d, e). Additionally, post hoc analysis revealed that at P30–32, WT males have higher activity modulation than KO males in AM/PM (*p* < 0.05) (Fig.5e). Our results show that genotype-dependent differences in dorsal stream activity emerge around or after P30–32 and then strengthen into adulthood. This suggests that there may be a window prior to the end of the visual-sensitive period for intervention to try to correct this dorsal stream deficit.

## Discussion

It is unclear how ASD-associated mutations alter cortical circuitry and contribute to the pathophysiology of ASD. Here, we used a mouse model of a *Cntnap2* null mutation to assess the impact of this mutation on the function of cortical circuitry. We found that a lack of *Cntnap2* (either Het or KO) in males results in decreased visually evoked activity in dorsal stream-associated HVAs, but in females, dorsal stream responses are similar among WT, Het, and KO mice. Loss of CNTNAP2 produces abnormal neuronal migration, decreased numbers of interneurons, and reduced cortical synchrony among other neuropathological abnormalities [15]. Our results shed light on the functional consequence at the level of an entire cortical region of these previously identified cellular-level effects.

Recently, altered sensory processing has been recognized as a key symptom for many individuals with ASD [1]. In addition to exhibiting tactile, somatosensory, and auditory sensitivities, individuals with ASD often show abnormalities in visual integration and global motion processing [33–36]. Based on these and similar findings, a “dorsal stream vulnerability” hypothesis has been proposed, wherein the late-maturing neural systems that underlie motion perception (dorsal stream) are more susceptible to early genetic or environmental effects than earlier maturing neural systems that underlie form perception (ventral stream) [37–39]. The finding that a lack of *Cntnap2* results in decreased visually evoked activity specifically in dorsal stream-associated visual areas in response to moving gratings is in line with this hypothesis. The observed decrease in dorsal stream HVA activity could provide a launching point for future studies seeking to examine the neuronal underpinnings of the human dorsal stream vulnerability hypothesis.

Male mice that were heterozygous for the *Cntnap2* mutation showed a reduction similar to KO males in dorsal-associated activity. The Het genotype is more similar to that found in humans, as complete KO is only seen in individuals with the rare neurodevelopmental disorder cortical dysplasia-focal epilepsy, of whom two thirds have an ASD diagnosis [11]. Previous studies using the same mouse line have not reported differences between WT and Het mice [15, 16, 40, 41]. Perhaps, the assay we present here can more sensitively detect subtle changes in cortical circuit functioning. If so, the assay could be useful in further studies of neurodevelopmental disorders [15, 41].

Prior studies of *Cntnap2* mice have not reported sex differences, and either use only one sex or pool both sexes in their results [15, 16, 40, 41]. In this context, our finding that females lacking *Cntnap2* show no change in visually evoked cortical activity is surprising. As noted previously, the incidence ratio of ASD is highly skewed towards males in high-functioning individuals with ASD [2, 3]. This imbalance could be due to similar mechanisms that underlie the sex-dependent effects of altered *Cntnap2* expression that we observed in mice. Human studies of SNPs and CNVs in *CNTNAP2* indicate there is a significant association between these mutations and ASD [7, 8]. One such study points to the significant association arising from affected males [8]. The authors interpret this finding by suggesting their female sample was too small due to the imbalance in incidence ratio by sex in ASD to adequately capture these mutations in females. However, the association they report in males could also suggest that males are more susceptible to the mutations they identified; consequently, in this interpretation, females carrying *CNTNAP2* mutations would not be often identified because they are more likely to be neurotypical. Our findings are supportive of the latter interpretation, showing that males are more susceptible to the effects of altered *Cntnap2* expression levels than females. This could be the result of females being able to carry additional mutational burden without being affected [42].

It is unusual that WT females showed relatively low levels of activity in dorsal stream-associated HVAs when raw V1 activity and ventral stream HVA activity was comparable to WT males. While we did not control for estrous cycle, given the number of individuals collected, it is likely that we sampled from both estrous and non-estrous individuals and the resulting data does not show a bimodal distribution or unusual variability. Further, a recent meta-analysis of 293 studies concluded that freely cycling females are no more variable than males [43]. Additionally, there was no difference in raw V1 activity, either by genotype or by sex. This implies that V1 activity is not affected by CNTNAP2, with WT males and females specifically showing no difference in raw V1 activity. This also argues that it was not simply an inability to detect visually evoked activity in female mice driving our findings. Furthermore, our ability to detect an effect in AM/PM in males argues against a floor effect, since dorsal stream HVA AL in females has a higher WT response level than AM/PM in males. From this, we conclude that the WT female data is an accurate representation of our colony.

In humans that go on to receive an ASD diagnosis, early neurodevelopment can be relatively typical prior to 2 years of age [44, 45]. In the mouse visual system, several different neurodevelopmental epochs have been identified. Prior to eye-opening around P13, connectivity in visual cortical circuitry is laid out by molecular genetic programs and spontaneous, internally generated patterns of activity [46–48]. After eye opening (~P13), visual cortical circuitry is rapidly sculpted by visual experience [49]. Beginning at P19, the classical critical period for ocular dominance plasticity refines cortical circuitry through P32 in mice [31]. These epochs in the mouse visual system development can place our developmental findings in a broader neurodevelopmental context and potentially provide a link to time points in which ASD is typically diagnosed. Our finding that genotype differences in visual responses in dorsal stream HVAs begin to emerge after P30 suggest that canonical critical period mechanisms for circuit refinement may not be driving the deficits observed in adulthood. Much is still unknown with regards to the development of HVAs; it is possible that dorsal stream HVAs in mice develop after the canonical critical period, contributing to the development of the deficit we observed. Future studies examining the early function and development of these HVAs as well as the effects of genetic and environmental manipulations on their development will greatly contribute to the field.

## Conclusions

By identifying how a non-syndromal ASD-linked mutation alters cortical development and cortical functioning, we have begun to understand how genetic factors perturb developing cortical circuitry to increase risk of developing ASD. This ISOI-based approach can provide analogous and complementary data to human functional imaging studies, for parallel human-mouse model investigations for identifying biomarkers, and investigating mechanisms that underlie neurodevelopmental disorders.

## Additional file

Additional file 1: Figure S1. Example retinotopy maps for male and female mice. A sample elevation and azimuth maps from WT, Het, and KO male mice used in the adult male experiments. White lines denote ROI boundaries between cortical visual areas. AM and PM are not separated in Het and KO due to lack of clear boundaries. Scale bar, 1 mm. B Sample elevation and azimuth maps from WT, Het, and KO female mice used in the adult female experiments. White lines denote ROI boundaries between cortical visual areas. AM and PM are not separated in Het and KO due to lack of clear boundaries. Scale bar, 1 mm. Regions V1, LM, LI, AL, and RL were consistently identified with high fidelity, regardless of genotype or sex. (EPS 23756 kb)

## Abbreviations

AL: Anterolateral area
AM: Anteromedial area
ASD: Autism spectrum disorders
CNTNAP2: Contactin-associated protein-like 2
Het: Heterozygous
HVA: Higher visual area
ISOI: Intrinsic signal optical imaging
KO: Knock-out
LI: Laterointermediate area
LM: Lateromedial area
PM: Posteromedial area
RL: Rostrolateral area
V1: Primary visual cortex
WT: Wild type
